# The group-based social skills training SOSTA-FRA in children and adolescents with high functioning autism spectrum disorder - study protocol of the randomised, multi-centre controlled SOSTA - net trial

**DOI:** 10.1186/1745-6215-14-6

**Published:** 2013-01-07

**Authors:** Christine M Freitag, Hannah Cholemkery, Leyla Elsuni, Anne K Kroeger, Stephan Bender, Cornelia Ursula Kunz, Meinhard Kieser

**Affiliations:** 1 Department of Child and Adolescent Psychiatry, Psychosomatics and Psychotherapy, University of Frankfurt, 60528, Frankfurt, Germany; 2 Department of Child and Adolescent Psychiatry, Psychosomatics and Psychotherapy, University of Dresden, 01307, Dresden, Germany; 3 Institute of Medical Biometry and Informatics, University of Heidelberg, 69120, Heidelberg, Germany

**Keywords:** Social skills training, Neural function, Moderating factors, Autism spectrum disorder

## Abstract

**Background:**

Group-based social skills training (SST) has repeatedly been recommended as treatment of choice in high-functioning autism spectrum disorder (HFASD). To date, no sufficiently powered randomised controlled trial has been performed to establish efficacy and safety of SST in children and adolescents with HFASD. In this randomised, multi-centre, controlled trial with 220 children and adolescents with HFASD it is hypothesized, that add-on group-based SST using the 12 weeks manualised SOSTA–FRA program will result in improved social responsiveness (measured by the parent rated social responsiveness scale, SRS) compared to treatment as usual (TAU). It is further expected, that parent and self reported anxiety and depressive symptoms will decline and pro-social behaviour will increase in the treatment group. A neurophysiological study in the Frankfurt HFASD subgroup will be performed pre- and post treatment to assess changes in neural function induced by SST versus TAU.

**Methods/design:**

The SOSTA – net trial is designed as a prospective, randomised, multi-centre, controlled trial with two parallel groups. The primary outcome is change in SRS score directly after the intervention and at 3 months follow-up. Several secondary outcome measures are also obtained. The target sample consists of 220 individuals with ASD, included at the six study centres.

**Discussion:**

This study is currently one of the largest trials on SST in children and adolescents with HFASD worldwide. Compared to recent randomised controlled studies, our study shows several advantages with regard to in- and exclusion criteria, study methods, and the therapeutic approach chosen, which can be easily implemented in non-university-based clinical settings.

**Trial registration:**

ISRCTN94863788 – SOSTA – net: Group-based social skills training in children and adolescents with high functioning autism spectrum disorder.

## Background

Autism spectrum disorders (ASD) are characterized by qualitative impairments in social interaction, reciprocal communication, and by stereotyped, repetitive behaviours. ASD comprise International Classification of Diseases, version 10 (ICD-10) diagnoses autism (F84.0), Asperger syndrome (F84.5), and atypical autism (F84.1), respective Diagnostic and Statistical Manual of Mental Disorders IV, text revision (DSM-IV TR) diagnoses autism (299.00), Asperger’s disorder (299.80) and pervasive developmental disorder-not otherwise specified (PDD-nos) (299.80). Recent epidemiological studies estimated a prevalence > 1% for all ASD, with about 45% showing an intelligence quotient (IQ) > 70 [1,2]. The rate of comorbid psychiatric disorders, especially attention-deficit/hyperactivity disorder (ADHD), anxiety and depressive disorders is high [3,4].

The treatment of choice in verbal school-aged children and adolescents with ASD and language and cognitive abilities in the average range, referred to as high functioning ASD (HFASD), is behaviour-based therapy to improve communication and social interaction with peers and adults. Group-based social skills training (SST) has been advocated as the most efficient treatment option. However, there is a scarcity of randomised controlled studies (RCT) implementing treatment manuals [5].

Review articles have delineated the following therapeutic goals that should be achieved by SST with ASD individuals [5,6]: increase of social motivation and social initiations, improvement of appropriate social responding, reduction of interfering behaviours and promotion of skill generalisation. Effective interventions are predominantly based on social learning theory, framing complex social conventions as rules that can be learned by, for example, modelling age-appropriate social interaction skills or teaching social scripts for common situations, and successively practising social interaction within the group by, for example, role play, video-modelling and direct feedback, as well as outside the group. In addition, operant methods as differential reinforcement of response attempts and positive behaviours are adopted. Some intervention methods also specifically aim at improving social understanding, emotion recognition, perspective-taking (that is, theory of mind abilities), and executive functions, as these aspects are specifically impaired in ASD [7]. As the aspect of generalisation, that is, the transfer and appropriate use of an acquired skill in other situations, is crucial for any effective SST program for children and adolescents with HFASD, generalisation sessions are added in several interventions. These can consist of community activities, training/interacting with typically developing peers, specific social interaction homework, or change of therapists during therapy [6].

Five RCTs in school-age children and adolescents with HFASD, including at least 10 children in the treatment group and implementing manualised training have been published to date [8-12]. A summary of the study characteristics, inclusion criteria and outcome measures are shown in Table 1. All studies were performed with children aged 7 to 12 years old; none included adolescent children. The studies differ considerably with respect to inclusion criteria, randomisation procedure (often not reported), kind and duration of the intervention, and outcome measures.

**Table 1:** Characteristics of published randomised controlled trials on social skills training in high-functioning autism spectrum disorder (HFASD)

**Publication / comparison groups**	**Inclusion / exclusion criteria**	**Subjects, number; age; RP**	**Intervention**	**Manual**	**Outcome measures pre/post treatment**	**Results pre/post treatment**	**Effect size group x time #**
Beaumont and Sofronoff, 2008, Junior Detective Training Program / treatment versus wait-list control condition	Asperger syndrome; IQ > 85; no exclusion criteria reported	N = 49; 7.5-11 years; RP not described	4-weekly computer game training sessions (child and parents, 45 minutes) plus 8-weekly group therapy sessions with global targets (2 therapists / 3 children, 75 minutes); parallel 8-weekly parent trainings; teacher handouts	Yes	No primary outcome measure		
					SSQ-P (PR)	*P* < 0.001	η^2^ = 0.37
					ERSSQ-P (PR)	*P* < 0.001	
					Child measures:		
					Emotion recognition	ns	
					Knowledge of emotion management strategies	*P* < 0.001	
					Treatment effect was maintained for 5 months (parent ratings)		
DeRosier *et al*., 2011 S.S.GRIN-HFA / treatment versus traditional S.S.GRIN	Autism, Asperger syndrome, PDD-nos; IQ > 85; exclusion of children with CBCL-aggression subscale > 70	N = 55; 8-12 years; RP not described	S.S.GRIN-HFA: 15 group sessions of 60 minutes, and 4 parent training sessions; traditional S.S.GRIN: 10 group sessions of 60 minutes (2 therapists/group)	Yes	No primary outcome measure		
					Parent report: SRS – subscales	*P* < 0.05	d: 0.68 - 0.94
					ALQ	*P* < 0.05	d = 0.75
					Child report:		
					Social dissatisfaction		
					questionnaire	ns	
					Social self-efficacy scale	ns	
Frankel *et al*., 2010 Children’s Friendship Training” / treatment versus wait-list control condition	Autism spectrum disorder (ADI-R, ADOS); verbal IQ > 60; child has to be able to switch topics in conversation and knows several play rules; attending regular classroom without individual support; exclusion criteria: psychotropic medication; neurological or medical disorder	N = 76 randomised, n = 68 completed training (58 boys, 10 girls); 7 - 12 years; RP: random.org	12 weeks of CFT within classes conducted by the UCLA friendship program; 12 session of 60 minutes for the child; 12 parallel sessions of 60 minutes for parents parents support homework and generalisation (i.e. arranging play dates)	Yes	No primary outcome measure		
					Child report:		
					The loneliness scale	*P* < 0.025	
					Piers-Harris self-concept scale	*P* < 0.025	
					parent report:	2 subscales *P* < 0.001	
					QPQ-P (5 subscales)		
					SSRS (4 subscales)	1 subscale *P* < 0.05	
					Teacher report: PEI treatment effect was maintained for 3 months (parent ratings)	ns	
Koenig *et al*., 2010 / treatment versus wait-list control condition	Pervasive developmental disorder (ADOS, SCQ, PDD-BI) full scale IQ >= 70; exclusion criteria: severe aggressive behaviour, self-injury, oppositional behaviour	N = 44; 8-11 years (34 boys, 10 girls); RP: by external faculty member	16 group therapy sessions of 75 minutes (2 therapists / 4-5 children, 2 peer tutors); after 3^rd^ session: individual treatment plan for each child (pro-social behavioural objectives; strategies)	Yes	Primary outcome measure: CGI-I	*P* = 0.001	
					Secondary outcome measure: Prosocial Index of the Social Competence Inventory (PR)	ns	
Lopata *et al*., 2010 / treatment versus wait-list control condition	Autism, Asperger Syndrome, PDD-nos IQ > 70, verbal IQ > 80; no exclusion criteria reported	N = 36; 7-12 years; RP not described	Summer training program: 5 weeks with 5 × 70 minutes treatment cycles/day and 3 therapists/6 children; parents: 1/week parent training of 90 minutes	Yes	Set of primary outcome measures; child measures:	One-tailed tests	
					social skills knowledge	*P* < 0.001	d = 1.27
					DANVA-2	ns	d = 0.53
					CASL	*P* < 0.001	d = 0.39
					parent ratings:		
					ASC	*P* = 0.006	d = 0.58
					SRS	*P* = 0.003	d = 0.63
					BASC-2-PRS: withdrawal	*P* < 0.001	d = 1.06
					BASC-2-PRS: social skills	ns	d = 0.37

ADI-R, Autism Diagnostic Interview-revised; ADOS, Autism Diagnostic Observation Schedule; ALQ, Achieved Learning Questionnaire; ASC, Adapted Skillstreaming Checklist; BASC-2-PRS, Behavior Assessment System for Children, 2^nd^ edition, parent rating scales; CASL, Comprehensive Assessment of Spoken Language; CBCL, Child Behavior Checklist; CFT, Children’s Friendship Training; CGI-I, Clinical Global Impressions Scale Improvement Item; DANVA-2, Diagnostic Analysis of Nonverbal Accuracy 2; ERSSQ, Emotion Regulation and Social Skills Questionnaire; ns, not significant; PDD-nos, Pervasive Developmental Disorder-not otherwise specified; PDD-BI, Pervasive Developmental Disorders Behaviour Inventory; PEI, The Pupil Evaluation Inventory; PR, parent report; QPQ, Quality of Play Questionnaire; RP, randomisation procedure; SCQ, Social Communication Questionnaire; SRS, Social Responsiveness Scale; SSQ-P, Social Skills Questionnaire, parent version; SSQ-T, Social Skills Questionnaire, teacher version; SSRS, Social skills rating system; TPSS, Teacher Perception of Social Skills.

# Effect sizes are shown were reported.

One study implemented a very intensive summer treatment programme over 5 weeks [12], and one was based within school settings [10]. One study aimed at targeting individual child-specific aims within a group-based setting [11]. The two remaining studies used the classical format of highly structured social skills training with weekly sessions for the children and additional parent training sessions of varying intensity [8,9].

With respect to inclusion criteria, both latter studies only included children with IQ > 85, whereas the other studies also included less cognitively able children with IQ > 60 or IQ > 70. Most studies did not report specific inclusion or exclusion criteria apart from the diagnosis of ASD, which, however, was not specifically confirmed by a standardised assessment prior to start of therapy in all studies. As ASD are heterogeneous disorders with a high rate of psychiatric and medical comorbidity and a variable course, not reporting standardised diagnostic assessment of ASD before start of the intervention, and not reporting psychiatric respective medical comorbid disorders are strong limitations of the published studies. In addition, only one study mentioned the use of psychotropic medication as an exclusion criterion [10], whereas the other studies did not report any information on the use of psychotropic medication. As the additional use of psychotropic medication is a major possible confounding factor, this is another limitation of most studies.

With respect to outcome measures, all studies implemented different parent rating scales for assessing social skills, which showed a positive effect of the respective training in all studies. Most studies did not report a primary outcome measure, but used several outcomes simultaneously. Effect sizes were not reported in all studies, and the long-term effect after 3 or 5 months of therapy was only assessed in two studies [8,10]. Where reported, effect sizes differed strongly by outcome measure between small and large effects. A few studies implemented child-based measures, which showed less strong or no effects compared to parent ratings.

Taken together, the previous randomised controlled studies on social skills training showed a medium to large effect on parent-reported social skills in children with ASD directly after the end of therapy, but suffer from several methodological and practical limitations. Thus, the present SOSTA-net trial aimed at overcoming several of these limitations by defining clear inclusion and exclusion criteria, one primary outcome and several secondary outcome measures assessing immediate and long-term effects of the therapy after 3 months, using a specific randomisation procedure, and also including adolescents and young adults in the trial. In addition, the large sample size (see below) will also allow the study of factors influencing therapy outcome, which is strongly relevant for clinical practice.

The first German group-based social skills training was developed at the Department of Child and Adolescent Psychiatry at JW Goethe University Hospital, Frankfurt am Main. A pre-post pilot study showed good acceptance of the intervention in individuals with HFASD and their parents, and was effective in improving social interaction with adults and peers [13]. As this first SST was designed as ongoing group training, and therefore did not contain a detailed manual for a limited number of group therapy sessions, in the current RCT, the effect of 12 sessions of a revised version, the manualised SOSTA-FRA programme is compared to treatment as usual (TAU), including wait-list, control condition. It is hypothesized that social responsiveness in children and adolescents with HFASD will improve more strongly with the group-based SOSTA-FRA training than by the TAU/wait-list.

As the effects of the previous SST studies varied across and within groups, it also is relevant to explore factors associated with treatment outcome. Well-established positive predictive factors are IQ and language ability. Less well-established influencing factors are severity of autistic symptoms in the three core domains; age, gender, psychiatric comorbid disorders, genetic risk factors, and medication effects [5,13,14]. These will be assessed in an exploratory fashion in the present study. In addition, the effect of SST on underlying brain mechanisms is examined by the electroencephalographic response to implicit and explicit facial emotion and biological motion recognition as correlates of social brain function.

## Methods/design

The SOSTA-net trial is designed as a prospective, randomised, controlled, multicentre trial, including an intervention and a waiting-list control group. The intervention includes 12 highly structured, manualised (SOSTA-FRA manual), weekly group-based social skills training, each of 90 minutes duration. Five children or adolescents take part in the intervention group, and the number of skilled behavioural therapists (that is, those who have studied at university for a minimum of 5 years and have undergone > 2 years of additional psychotherapy training) in each group is two (child-therapist ratio = 5:2). In addition, three parent training sessions are scheduled at the beginning, middle and end of the group therapy sessions, both for the intervention and the control group. The wait-list control group receives the same social skills training after the end of trial, that is, after 6 months (3 months intervention and 3 months follow-up).

### Objectives and hypothesis

It is hypothesized that add-on group-based SST using the manualised treatment program SOSTA-FRA will result in improved social responsiveness and pro-social behaviour compared to TAU. The parent-rated Social Responsiveness Scale (SRS), obtained at baseline, directly after intervention, and at 3 months follow-up is the primary outcome measure of interest (see below).

Several additional aspects with regard to secondary outcome measures will be studied: A second scale, the Strength and Difficulties Questionnaire (SDQ), assessing pro-social behaviour, total and peer-related problems, will be obtained from parents, and improvements in all three areas are expected. Also, it is expected, that social responsiveness (SRS), pro-social behaviour, total and peer-related problems (SDQ) as reported by teachers will be improved. These are measures of generalisation of the acquired social skills into the classroom. In addition, a positive effect on parent- and self-rated anxious and depressive symptoms is expected.

Variables that could possibly moderate treatment effect, including age, IQ, sex, comorbid disorders, ASD symptom severity, medication and common variants of genes related to social interaction will be explored. Exclusively in Frankfurt, changes in neural function during perception of social stimuli, measured by electroencephalograpy (EEG), will also be assessed pre- and post-therapy and at 3 months follow-up.

### Study centres

The trial is performed by close cooperation between six University Hospital Departments of Child and Adolescent Psychiatry, Psychosomatics and Psychotherapy in Germany, which provide a special outpatient service for individuals with ASD: Aachen, Frankfurt/Main, Homburg/Saar, Köln, Wuerzburg, and Mannheim. Data management, biometric support and statistical analyses are provided by the Institute of Medical Biometry and Informatics (IMBI), University of Heidelberg. Study monitoring is done by the Coordination Centre for Clinical Trials (KKS), University of Heidelberg.

### Participants‘ inclusion and exclusion criteria

A diagnosis of autism, Asperger syndrome or atypical autism (ICD-10) is essential to be included in the study. Diagnostic assessment is standardized according to ICD-10, by obtaining a detailed medical history, performing the Autism Diagnostic Interview-Revised (ADI-R), the Autism Diagnostic Observation Schedule (ADOS), and a standardized intelligence testing (HAWIK-IV, WIE). Patients’ incusion and exclusion criteria are listed in Table 2. Comorbid psychiatric disorders are diagnosed according to ICD-10 and DSM-IV TR by a German validated structured interview on psychiatric disorders in children, the Diagnostic interview on psychiatric disorders in children, German version (Kinder-DIPS) [15]. The sample comprises a group of children and adolescents with high-functioning autism, Asperger Syndrome, and atypical autism who are typical participants of SST in ASD. Inclusion and exclusion criteria with regard to other psychiatric disorders are broad, by only excluding individuals with schizophrenia, bipolar disorder, social phobia, obsessive-compulsive disorder, major depressive disorder with suicidal ideation, or any personality disorder as well as aggressive behaviour interfering with group therapy. This approach reflects the high rate of comorbid psychiatric disorders in ASD [4], which are likely to be relevant with respect to therapeutic outcome. Only disorders which require a more specific and different kind of psychopharmacotherapy and/or psychotherapy were excluded as comorbid disorders. Other frequent comorbid disorders, such as attention-deficit/hyperactivity disorder or oppositional behaviour as well as specific phobias or depressive episodes without suicidal ideation, were no reason for exclusion. They will be assessed in secondary analyses as possible moderating factors of therapy outcome. Similarly, inclusion criteria with regard to cognitive abilities were rather broad allowing children with IQ >=70 into the study. We will enrol 220 subjects into the clinical trial, that is, 110 subjects per treatment group. Only a single participation in the trial is allowed.

**Table 2:** Inclusion and exclusion criteria of the SOSTA-net trial

**Inclusion criteria**	Diagnosis of autism spectrum disorder (ICD-10: F84.0, F84.5, F84.1)
	Age 8-20 years at start of group therapy
	Informed consent
	No, or stable psychopharmacotherapy
	Children and parents are fluent in reading German to be able to understand instructions and fill in questionnaires
**Exclusion criteria**	IQ < 70 (full scale IQ according to HAWIK-IV/WIE)
	Psychiatric disorders: schizophrenia, bipolar disorder, social phobia, obsessive-compulsive disorder, major depressive episode with suicidal ideation, any personality disorder
	Aggressive behaviour interfering with group therapy
	Any neurological disorder (exception: well treated epilepsy)
	Other medical condition interfering with therapy
	Group-based SST during last 6 months prior to the study
	Participation in other clinical trials including observation period of competing trials

HAWIK-IV/WIE, Wechsler Scales for children, adolescents and adults with current German norms, measuring IQ; IQ, intelligence quotient.

### Criteria for withdrawal

Subjects can withdraw from the trial (1) at their own request or at the request of the legal representative; (2) if in the investigator’s opinion continuation of the trial would be detrimental to the subject’s well-being; (3) on admission to a psychiatric clinic; (4) if in the investigator’s opinion the realisation of the program is no longer possible due to the patient’s behaviour in group therapy sessions, for example, oppositional or aggressive behaviour directed against other group members or herself/himself. A change in pharmacotherapy is not a criterion for withdrawal from the trial.

### Prior and concomitant treatments

Relevant additional treatments administered to the subjects on entry to the trial or at any time during the trial are regarded as concomitant treatments und are documented on the appropriate pages of the case report form (CRF). TAU is allowed in the intervention as well as the control group. TAU includes stable psychopharmacotherapy, stable medication for chronic medical conditions not interfering with group therapy, individual language, psychomotor or behaviour therapy, personal support at school or family support. Kind and frequency of TAU in the treatment and control groups will be compared for non-random distribution in the statistical analysis.

Psychotropic medication is started or changed at least four weeks before randomisation and remains stable (mg/kg body weight) throughout the intervention and the three months follow-up of the study (with the exception of dose adjustment to body weight changes). The following psychotropic medication is allowed as single or combined treatment: SSRI, other antidepressants, typical or atypical neuroleptics, stimulants, atomoxetine, or mood stabilisers. In addition, stable medication for the treatment of chronic conditions such as allergies, asthma, epilepsy, enuresis, sleeping problems, and intermitting medication for acute upper respiratory infections and diarrhoea is allowed. Pharmacological treatment is documented at each time of assessment (T1-T5), and effects of psychotropic medication on treatment outcome will be explored in the statistical analysis.

Any individual intervention (for example, cognitive behavioural therapy, school-based intervention, occupational, language, psychomotor therapy) or family-based intervention is also allowed. Any additional treatment is documented in detail (kind of intervention, dose, frequency, etcetera).

### Intervention

SOSTA-FRA is manualised, structured cognitive-behavioural therapeutic and group-based social skills training for children and adolescents with ASD. It combines psycho-educational, experimental, and operant methods of teaching and practising social skills. The groups are lead by two trained behavioural psychotherapists. Twelve sessions, each of 90 minutes duration, are provided. Each session follows a standard sequence of activities: saying hello/greeting, opening round with group circuit and repetition of group-rules, session specific topic (which consists of the introduction of new skills followed by the respective training), group game, homework and closing round with feedback. The following social skills are taught, discussed, and practised within the 12-week curriculum: establishing group rules, using eye contact, introducing oneself to others, awareness and expression of feelings, non-verbal communication, politeness, listening, getting into contact with peers, having fun being part of a social group, apologising, conversational skills, negotiation, dealing with teasing/bullying, making friends, problem solving, self-regulation, impulse control, and strategies for coping with anger. Thus, SOSTA-FRA takes on the essential topics and methods that have been recommended for ASD-specific SST as delineated in the introduction section (aiming at increasing social motivation and social initiations, improvement of appropriate social responding, improvement of executive functions, reduction of interfering behaviours and promotion of skill generalisation by social learning and operant methods, as well as cognitive approaches). Every session includes homework to ensure transfer and generalisation of acquired skills. The last session includes a group activity outside the clinical setting. SOSTA-FRA implements a token program for the single session and for homework, and therapists provide extensive positive reinforcement of response attempts, of appropriate responses, and of use of acquired skills.

Accompanying the group-based intervention or during the waiting time of the control group, three parent training sessions, each of 90 minutes duration are additionally provided for the intervention and the control group. The aim of the parent training sessions is to ensure compliance, educating parents with regard to ASD in a general way, supporting parents to cope with their children, increase support between families, giving parents the opportunity to talk about current issues and aspects of their children, and to obtain the study questionnaires on a regular basis.

### Assessment

**Figure 1:**
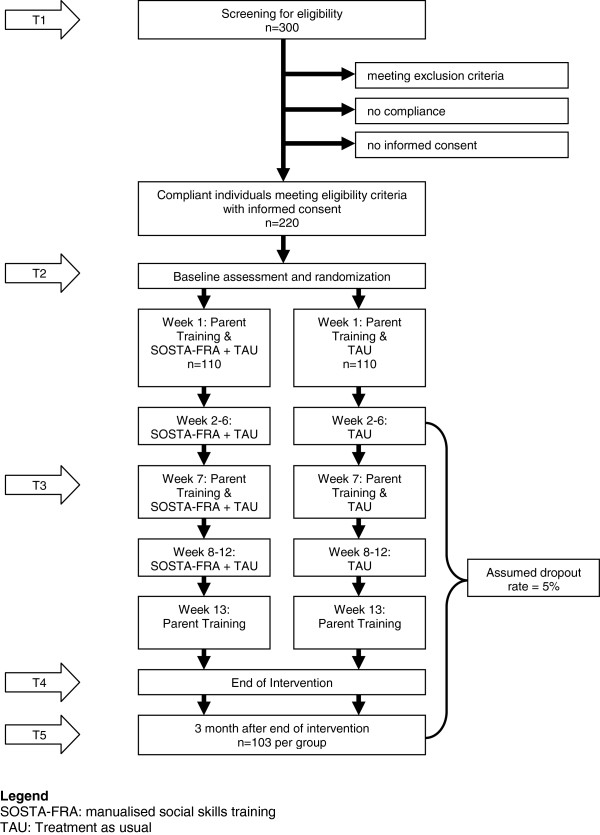
Trial Time Flow.

The trial time flow is shown in Figure 1. After verifying the diagnosis of HFASD and screening for eligibility (T1), ten individuals are included in an age-homogenous study group. The participants are then randomised to the intervention and control group, and the baseline assessment (T2) takes place. Following randomisation, the first parent training (both for parents of the children in the intervention and the control group) and the first SST group session of at least five individuals are performed. Mid-intervention assessment takes place after 6 weeks group training, parallel with the second parent training. This mid-intervention assessment (T3) aims at obtaining the primary and secondary outcome measures during the ongoing trial to get some information on individuals dropping out from the study before T4. One week after the last group SST session, the third parent training is provided and post assessment takes place (T4). To assess the stability of the therapy effects, the study also includes a follow-up (T5) measurement 3 months after finishing the SST.

### Outcome measures

The primary outcome measure is the parent-rated Social Responsiveness Scale (SRS) which was developed as a change-sensitive measure of social abilities [16,17]. It contains 65 items covering social responsiveness and interaction, communication and stereotyped behaviours in children and adolescents. Five subscales (social awareness, social cognition, social communication, social motivation and autistic mannerisms) and a summary score can be calculated. Change in the total raw score of the Social Responsiveness Scale (SRS) as assessed by the primary caretaker (PC) (a) between baseline (T2) and 12 weeks after baseline (T4) (primary endpoint), as well as (b) between baseline (T2) and 3 months after the end of the intervention (T5) (co-primary endpoint) is the primary outcome measure in this study. The parent-rated SRS has been chosen as the outcome measure in several studies on SST in HFASD [9,12,14].

The secondary outcome measures aim to differentially assess aspects of social interaction, psychopathology, depressive and anxious symptoms. All secondary outcome measures have been validated and are frequently used in clinical research in child psychology and psychiatry. Some of these secondary outcome measures have not been standardized for individuals above 16 or 18 years of age. However, as changes in raw scores are used as secondary outcome measures in this study, the reported scales are considered as valid measures. The following secondary outcome measures will be assessed directly after therapy and 3 months after the end of intervention:

1. Response to intervention: individual symptom reduction of at least 16 raw points (1 SD) in PC-rated SRS total score

2. Teacher (T)-rated SRS (change in total raw score and response to intervention, that is, reduction of at least 16 raw points in the T-rated SRS)

3. Change in the SRS subscale raw scores in PC, T-rated SRS

4. Change in PC-rated anxious-depressive symptoms – raw score as assessed by the child-behavior checklist (CBCL). The CBCL is a parent rating scale with 120 Likert (0 to 3)-coded items to assess psychopathology in different aspects of behaviour [18]. In this study, the anxious-depressive subscale raw score (14 items) is chosen, as individuals with HFASD often suffer from comorbid anxiety and depressive disorder, possibly related to their problems with social interaction

5. Change in total, pro-social behaviour and peer-related problems raw scores as assessed by the Strength and Difficulties Questionnaire (SDQ: PC, T-rated). The SDQ is a widely used, German-translated and validated instrument [19] and includes subscales of conduct, emotional, hyperactivity and peer-related problems, as well as pro-social behaviours

6. Change in self-reported depressive symptoms as assessed by the *Depressionsinventar für Kinder und Jugendliche* (DIKJ). The DIKJ is a self-reported scale for children and adolescents 8 to 16 years of age and contains 26 Likert (0 to 3)-coded items [20]

7. Qualitative information on motivation and satisfaction with therapy (self-assessment)

8. assessment of functional genetic variants and psychotropic medication as possible moderating variables of therapeutic effect;

9. change in electrophysiological brain activation during the processing of social stimuli. EEG based evoked potentials to emotional faces and Mu oscillations to biological motion patterns to study neural change induced by SST are obtained at T2, T4 and T5 at the Frankfurt study site, aiming at including 25 HFASD individuals from the treatment and 25 HFASD from the control group.

### Sample size calculation

The sample size calculation refers to the primary endpoint, change in SRS total score between baseline and 12 weeks after baseline, in the ITT population. The prior assumption for the treatment effect of the experimental intervention is based on the results of Tse *et al*. (2007) with a treatment mean difference of 11 points and SD of 28 points. It is assumed that the mean SRS total score does not change for the control group and that the same SD as in the experimental intervention holds true. With a two-sided significance level of α = 5% and a power of 1-β = 80%, a sample size of 206 (2 × 103) is required to detect this difference with a two-sample *t*-test. It can be expected that application of an analysis of covariance as defined for the evaluation (see below) will reduce the SD, thus increasing the actual power of the study. Based on the observations made in the study of Tse *et al*. (2007), the dropout rate during the intervention is assumed to be about 5%. Therefore, 220 individuals will be randomised in total, to obtain at least 206 evaluable patients. On the basis of the information from the clinical study centres, it is expected that about 25% of the screened patients cannot be included in the study due to violation of the inclusion/exclusion criteria. Hence, 300 patients will be assessed for eligibility. Sample size calculation was done using nQuery Advisor 7.0 [21].

### Methods against bias

To randomly allocate influencing factors and to avoid selection bias, patients are randomly assigned by centralised randomisation to each group (intervention group and wait-list/TAU control group). An internet-based randomisation system (http://randomizer.at) is used. The randomisation is done in an allocation ratio of 1:1 with variable block length and is stratified for each participating centre.

To ensure that all participating centres will implement the SST and parent training in a very comparable way across centres, all therapists are trained in the SOSTA-FRA program before the beginning of the study in their centre. Further, all therapists are supplied with a detailed written manual including all performance rules. Regular telephone supervision and meetings are provided. In addition, one therapy session of each intervention group is videotaped and analysed for compliance with the manual by independent raters at the Frankfurt study centre. Reliability training in diagnostic measures has been performed before the start of the study.

As the proposed study is a behavioural therapy intervention study, blinding of participants as well as therapists or parents is not possible. Teachers are blinded to intervention. Therefore, a possible bias of the SRS-parent scores can be detected by comparing it to the SRS-teacher scores. The teacher scores, however, will not be used as primary outcome measure, as it is to be expected from previous studies [22] that return of teacher questionnaires will only be around 50 to 60%. Independent clinical on-site monitoring to ensure patient safety and integrity of the clinical data in adherence to the study protocol focuses on source data documentation, correctness of data, and adherence to trial procedures, for example, randomisation and treatment.

### Statistical analysis

Confirmatory analysis is performed for the ITT population, and is defined on the basis of the ITT principle. An additional per-protocol analysis will be conducted, which will include all patients without major protocol violations. Two primary endpoints are assessed in confirmatory analysis, namely the change in SRS total score between baseline and 12 weeks after baseline (effect at the end of therapy) and the change in SRS total score between baseline and three months after the end of therapy (maintenance effect). To ensure control of the multiple type I error rate at 5%, a hierarchical test procedure will be applied [23]: The first null hypothesis to be tested states that the change in SRS total score between baseline and 12 weeks after baseline is equal for both groups. If this hypothesis can be rejected at the two-sided significance level of 5%, the second null hypothesis stating that the change in SRS total score between baseline and three months after the intervention is equal in both groups is tested at the two-sided level 5%. If the first null hypothesis cannot be rejected, the second null hypothesis referring to the co-primary endpoint is also accepted.

Both null hypotheses will be tested by analysis of covariance with the covariates baseline SRS total score, age, IQ, and centre. Gender and medication effects were not observed in previous studies [14], and hence these variables are not included in the model of the primary analysis. Missing values with respect to post-baseline primary outcomes will be handled by application of the mixed-effects model for repeated measures (MMRM) approach that turned out to show favourable characteristics in terms of the type I error rate, power, and bias of estimates as compared to alternative methods dealing with missing values, such as last-observation-carried-forward (LOCF) [24-26].

Descriptive methods will be used for the analysis of the secondary outcomes, including calculation of appropriate summary measures of the empirical distribution as well as calculation of descriptive two-sided *P*-values. A special focus of the exploratory analysis will be with respect to the time course of the primary as well as the secondary endpoints. Additionally, sensitivity analyses will be conducted for different populations (per-protocol population, appropriate subgroups) and applying alternative imputation techniques (such as LOCF) for missing values. Further exploratory analyses will be performed to identify intervention effects in subgroups and potential prognostic factors (including genetic variants) for an intervention effect. Graphical methods will be applied to visualise the findings of the study. The safety analysis includes calculation and comparison of frequencies and rates of adverse and serious adverse events (SAEs) reported in the two intervention groups. All analyses will be done using the latest SAS version (9.1 or higher).

### Safety aspects

An independent Data and Safety Monitoring Board (DSMB) is established, involving two independent clinical experts and one biometrician for monitoring the progress of the trial and ensure adherence to protocol. Safety parameters are all SAEs and adverse events (AEs) reported by the subject or PC, or detected by the local investigator, that occur during and up to three months after treatment. All noticeable problems must be documented in the CRF. Even though the character of the intervention as a behavioural therapy makes SAEs extremely unlikely, the Ethics Committee and DSMB will be informed in the case of SAEs within 24 hours after the SAE becomes known.

### Ethical issues

The procedures set out in the trial protocol pertaining to conduct, evaluation and documentation of this trial is designed to ensure that all persons involved in the trial comply with Good Clinical Practice (GCP) and the ethical principles described in the latest accepted version of the Declaration of Helsinki in Germany. The trial is carried out in keeping with local legal and regulatory requirements, although German Drug Law (AMG) and Medical Device Law (MPG) are not applicable. Each site’s principal investigator ensures that all persons assisting with the trial are adequately informed about the protocol, any amendments to the protocol, the trial treatments, and their trial-related duties and functions. Before the first subject has been enrolled, all ethical and legal requirements have been met. Study protocol, patient information and the respective consent form were approved by the responsible Ethical Committees before the start of the trial. The study protocol was first ethically reviewed by the ethical committee of the Medical Faculty, Goethe Universität, Frankfurt am Main, Germany, on 30 March 2010 (No. 57/10). Subsequent approval of this vote was done by the ethical committees of the Medical Faculties in Aachen (27 August 2010), Köln (1 September 2010), Mannheim (7 September 2010), Würzburg (25 October 2010), and the Ärztekammer des Saarlandes, Germany (26 May 2010). Parents and participants are informed individually about the study and need to consent separately to the three parts of the study (intervention, genetics, and neurophysiology). Participants are allowed to take part only in the intervention study if they do not agree to the genetic or neurophysiological part of the study. Throughout the trial, subjects are pseudonymised. Authorized persons (clinical monitors) regularly control adherence to protocol. During on-site visits, they inspect subject-related data to ensure adherence to data protection laws and correctness and completeness of data. As TAU is allowed for the treatment and the control group, and as the control group can take part in the training after completion of the trial, the treatment and control group experience no disadvantage related to clinical intervention by taking part in the trial.

## Discussion

This study is currently one of the largest trials worldwide on SST in children and adolescents with HFASD. When the study was planned in 2008, no RCTs on SST in HFASD had been published in an English, or German, scientific peer-reviewed journal. Since then, the studies shown in Table 1 have been published. Compared to these studies, our study shows several advantages with regard to the following aspects: (1) sample with specific inclusion and exclusion criteria; (2) study methods; and (3) therapeutic approach.

With regard to the ASD sample, not only the diagnostic criteria for ASD are obtained by standardised instruments, but also an additional standardised diagnostic interview is performed to assess psychiatric comorbid disorders in the study participants. Only psychiatric disorders that necessitate a different kind of therapeutic management are excluded as comorbid disorders. This reflects the current epidemiological findings on the high rate of psychiatric comorbid disorders in ASD [4], and allows secondary analyses on the kind and rate of comorbid psychiatric disorders and their influence on therapeutic outcome. Going beyond previous RCTs, not only children but also adolescents are included, and the study manual provides specific age-appropriate training material. Also, children with IQ >= 70 (compared to IQ > 85) are allowed into the trial, which reflects the clinical need of many children with ASD. These inclusion criteria are rather broad and may result in a somewhat smaller effect size of the intervention in the full group compared to the recently published trials, but also will allow probing for factors influencing therapy outcome. Similarly, stable medication is allowed in this trial, reflecting clinical practices, and again allowing testing of the influence of the combined treatment on outcome in secondary analyses.

With regard to study methods, and similar to previous studies, parent rating (here: the parent- rated social responsiveness assessed by SRS) was chosen as the primary outcome measure. This is a critical aspect, as parents and their children cannot be blinded with regard to the intervention. Teachers are blind to the allocation of the ASD individuals to the intervention or control group. Thus, the present study also aims to obtain as many teacher-reported SRS data as possible. However, due to the well known difficulties in obtaining complete teacher ratings compared to parent ratings, the teacher-rated SRS was not chosen as a primary outcome measure. In addition to the primary outcome measure, several additional secondary outcome measures tapping ASD-specific behaviour and comorbid anxious and depressive symptoms, as well neural functional change induced by therapy, are clearly going beyond the currently published RCTs on SST in ASD. The automated online randomisation procedure is clearly defined and reflects the study design with respect to the multi-centre study and the group-based randomisation. The statistical analysis also takes the different study centres into account. A strong advantage of the current study is the 3 months follow-up assessment, providing some information on long-term effects of group-based SST in HFASD.

Finally, the therapeutic approach chosen, combining child-based SST with parent training to increase the children’s compliance with the training is a setting that can be easily implemented in non-university clinical settings. Compared with the published RCTs (Table 1), therapeutic frequency is fairly similar with 12-weekly sessions of 90 minutes of SST. The only exception to the weekly training sessions was in the very intensive summer camp treatment program by Lopata *et al*. (2010), which provides many more therapy hours, but might be less cost-effective compared to weekly SST programs that can also be provided throughout the year. In addition to the traditional SST in ASD focusing on social rule-based learning, social understanding and practising of social skills, the SOSTA-FRA manual also includes executive function training, anger control strategies, and training of behavioural and cognitive flexibility overcoming stereotyped behaviour and intense special interests interfering with social interaction. Thereby, it addresses the broad range of everyday difficulties in children and adolescents with ASD.

In conclusion, this large multi-centre RCT on the manualised SOSTA-FRA program addresses a strong clinical need and will provide further insight into the efficacy of SST, its moderating factors, and the 3-month long-term effects of SST in individuals with HFASD.

## Trial status

Patient recruitment started on 20 May 2010 and will be finished in March 2013. Currently (October 2012), 204 patients have been randomised.

## Abbreviations

ADI-R: Autism Diagnostic Interview-revised; ADOS: Autism Diagnostic Observation; AE: Adverse event; AMG: German Drug Law (*Arzneimittelgesetz*); ASD: Autism spectrum disorder; CBCL: Child behaviour checklist; CRF: Case report form; DFG: German Research Foundation (*Deutsche Forschungsgemeinschaft*); DIK: Depression inventory for children and adolescents; DSM-IV TR: Diagnostic and Statistical Manual of Mental Disorders IV, text revision; DSMB: Data and Safety Monitoring Board; EC: Ethics Committee; EDTA: Ethylenediaminetetraacetic acid; FSI: First subject in; FSIQ: Full scale IQ; GCP: Good Clinical Practice; HAWIK-IV: Wechsler Intelligence Scale for children, version IV (German version); HDMS: Hospital Data Management System; HFASD: High-functioning autism spectrum disorder; ICH: International Conference on Harmonization of Technical Requirements for Registration of Pharmaceuticals for Human Use; IE: Individual estimation; IMBI: Institute of Medical Biometry and Informatics; IQ: Intelligence quotient; IRB: Institutional Review Board; ISF: Investigator site file; ITT: Intention to treat; Kinder-DIPS: Diagnostic interview on psychiatric disorders in children (German version); KKS: Coordination Centre for Clinical Trials (*Koordinierungszentrum für Klinische Studien*); LOCF: Last observation carried forward; LSI: Last subject in; LSO: Last subject out; MMRM: Mixed-effects model for repeated measures; MPG: Medical device law; O: Others; PC: Primary caretaker; PI: Primary investigator; PR: Patient registry; RCT: Randomised controlled trial; SAE: Serious adverse event; SDQ: Strength-and-Difficulties Questionnaire; SDV: Source Data Verification; SES: Socio-economic status; SOP: Standard operating procedure; SRS: Social Responsiveness Scale (German version); SST: Social Skills Training; T: Teacher; T1, 2, 3, 4, 5: Timepoint 1, 2, 3, 4, 5; TAU: Treatment as usual; TMF: Trial master file; WIE: Wechsler Intelligence Scale for adults (German version).

## Competing interests

The authors declare that they have no competing interests.

## Authors’ contributions

CMF designed and planned the SOSTA-net trial. HC and CMF wrote the therapy manual. CMF, HC and LE are responsible for the organisation of the trial, and CMF is the study PI. SB designed and AK performs the neurophysiological study at the Frankfurt site. CUK and MK were involved in the design of the study, performed the sample size calculation, and planned the statistical analysis. CMF, HC, and MK wrote a first draft of the present article. All authors read and corrected the first draft, and approved the final manuscript.

## References

[B1] Baird G Simonoff E Pickles A Chandler S Loucas T Meldrum D Charman T Prevalence of disorders of the autism spectrum in a population cohort of children in South Thames: the Special Needs and Autism Project (SNAP) Lancet 2006 368 210 215 10.1016/S0140-6736(06)69041-7 16844490

[B2] Baron-Cohen S Scott FJ Allison C Williams J Bolton P Matthews FE Brayne C Prevalence of autism-spectrum conditions: UK school-based population study Br J Psychiatry 2009 194 500 509 10.1192/bjp.bp.108.059345 19478287

[B3] Leyfer OT Folstein SE Bacalman S Davis NO Dinh E Morgan J Tager-Flusberg H Lainhart JE Comorbid psychiatric disorders in children with autism: interview development and rates of disorders J Autism Dev Disord 2006 36 849 861 10.1007/s10803-006-0123-0 16845581

[B4] Simonoff E Pickles A Charman T Chandler S Loucas T Baird G Psychiatric disorders in children with autism spectrum disorders: prevalence, comorbidity, and associated factors in a population-derived sample J Am Acad Child Adolesc Psychiatry 2008 47 921 929 10.1097/CHI.0b013e318179964f 18645422

[B5] Williams White SW Koenig K Scahill L Social skills development in children with autism spectrum disorders: a review of the intervention research J Autism Dev Disord 2007 37 1858 1868 10.1007/s10803-006-0320-x 17195104

[B6] Rao PA Beidel DC Murray MJ Social skills interventions for children with Asperger's syndrome or high-functioning autism: a review and recommendations J Autism Dev Disord 2008 38 353 361 10.1007/s10803-007-0402-4 17641962

[B7] Solomon M Goodlin-Jones BL Anders TF A social adjustment enhancement intervention for high functioning autism, Asperger's syndrome, and pervasive developmental disorder NOS J Autism Dev Disord 2004 34 649 668 10.1007/s10803-004-5286-y 15679185

[B8] Beaumont R Sofronoff K A multi-component social skills intervention for children with Asperger syndrome: the Junior Detective Training Program J Child Psychol Psychiatry 2008 49 743 753 10.1111/j.1469-7610.2008.01920.x 18503531

[B9] DeRosier ME Swick DC Davis NO McMillen JS Matthews R The efficacy of a Social Skills Group Intervention for improving social behaviors in children with High Functioning Autism Spectrum disorders J Autism Dev Disord 2011 41 1033 1043 10.1007/s10803-010-1128-2 21042870

[B10] Frankel F Myatt R Sugar C Whitham C Gorospe CM Laugeson E A randomized controlled study of parent-assisted Children's Friendship Training with children having autism spectrum disorders J Autism Dev Disord 2010 40 827 842 10.1007/s10803-009-0932-z 20058059 PMC2890979

[B11] Koenig K White SW Pachler M Lau M Lewis M Klin A Scahill L Promoting social skill development in children with pervasive developmental disorders: a feasibility and efficacy study J Autism Dev Disord 2010 40 1209 1218 10.1007/s10803-010-0979-x 20204689

[B12] Lopata C Thomeer ML Volker MA Toomey JA Nida RE Lee GK Smerbeck AM Rodgers JD RCT of a manualized social treatment for high-functioning autism spectrum disorders J Autism Dev Disord 2010 40 1297 1310 10.1007/s10803-010-0989-8 20232240

[B13] Herbrecht E Poustka F Birnkammer S Duketis E Schlitt S Schmotzer G Bolte S Pilot evaluation of the Frankfurt Social Skills Training for children and adolescents with autism spectrum disorder Eur Child Adolesc Psychiatry 2009 18 327 335 10.1007/s00787-008-0734-4 19165532

[B14] Tse J Strulovitch J Tagalakis V Meng L Fombonne E Social skills training for adolescents with Asperger syndrome and high-functioning autism J Autism Dev Disord 2007 37 1960 1968 10.1007/s10803-006-0343-3 17216559

[B15] Unnewehr S Schneider S Margraf J Kinder-DIPS - Diagnostisches Interview bei psychischen Störungen im Kindes- und Jugendalter 2009 Berlin: Springer 10.1024/1422-4917//a000247 23988834

[B16] Constantino JN Davis SA Todd RD Schindler MK Gross MM Brophy SL Metzger LM Shoushtari CS Splinter R Reich W Validation of a brief quantitative measure of autistic traits: comparison of the social responsiveness scale with the autism diagnostic interview-revised J Autism Dev Disord 2003 33 427 433 10.1023/A:1025014929212 12959421

[B17] Bölte S Poustka F Skala zur Erfassung sozialer Reaktivität 2008 BernHuber

[B18] Arbeitsgruppe Deutsche Child Behavior Checklist: *Deutsche Bearbeitung der Child Behavior Checklist (CBCL/4-18) - Einführung und Anleitung zur Handauswertung, 2. Auflage mit deutschen Normen*. Köln: author’s print 1999

[B19] Rothenberger A Becker A Erhart M Wille N Ravens-Sieberer U Psychometric properties of the parent strengths and difficulties questionnaire in the general population of German children and adolescents: results of the BELLA study Eur Child Adolesc Psychiatry 2008 17 99 105 19132309 10.1007/s00787-008-1011-2

[B20] Stiensmeier-Pelster J Schürmann M Duda K DIKJ - Depressionsinventar für Kinder und Jugendliche 2000 Göttingen: Hogrefe

[B21] Elashoff JD nQuery Advisor Version 7 User's Guide 2007 Los Angeles: Statistical Solutions

[B22] Laugeson EA Frankel F Mogil C Dillon AR Parent-assisted social skills training to improve friendships in teens with autism spectrum disorders J Autism Dev Disord 2009 39 596 606 10.1007/s10803-008-0664-5 19015968

[B23] Maurer M Hothorn L Lehmacher W Stuttgart VJ: Fischer Multiple comparisons in drug clinical trials and preclinical assays: a priori ordered hypothesis Biometrie in der chemisch-pharmazeutischen Industrie. Volume 6 1995 3 12

[B24] Mallinckrodt CH Lane PW Schnell D Peng Y Mancuso JP Recommendations for the primary analysis of continuous endpoints in longitudinal clinical trials Drug Inf J 2008 42 303 319 10.1177/009286150804200402

[B25] Lane P Handling drop-out in longitudinal clinical trials: a comparison of the LOCF and MMRM approaches Pharm Stat 2008 7 93 106 10.1002/pst.267 17351897

[B26] Siddiqui O Hung HM O'Neill R MMRM vs. LOCF: a comprehensive comparison based on simulation study and 25 NDA datasets J Biopharm Stat 2009 19 227 246 10.1080/10543400802609797 19212876

